# Three-Dimensional Study of Polymer Composite Destruction in the Early Stages

**DOI:** 10.3390/polym15020276

**Published:** 2023-01-05

**Authors:** Vadim Levin, Yulia Petronyuk, Igor Artyukov, Inna Bukreeva, Alexander Malykhin, Elena Longo, Lorenzo D’Amico, Konstantinos Giannoukos, Giuliana Tromba

**Affiliations:** 1 Laboratory of Acoustic Microscopy, N.M. Emanuel Institute of Biochemical Physics, Russian Academy of Sciences, 119334 Moscow, Russia; 2 Scientific and Technological Center of Unique Instrumentation, Russian Academy of Sciences, 117342 Moscow, Russia; 3 X-ray Optics Laboratory, P.N. Lebedev Physical Institute, Russian Academy of Sciences, 119991 Moscow, Russia; 4 Department of Control Systems of Robotic Complex, Scientific and Educational Center “Robotics”, Bauman Moscow State Technical University, 105005 Moscow, Russia; 5 Elettra Sincrotrone Trieste, Area Science Park, Basovizza, 34149 Trieste, Italy; 6 UMS 3360 DMEX-Centre for X-ray Imaging, Halle Technologique, UFR Sciences et Techniques, Avenue de L’Université, BP 1155, 64013 Pau, France

**Keywords:** carbon fiber, CFR polymer, X-ray, computed tomography, fracture dynamics

## Abstract

The investigation of destruction processes in composite materials is a current problem for their structural application and the improvement of their functional properties. This work aimed to visualize structural changes induced in layered carbon fiber reinforced plastics (CFRP) with the help of synchrotron X-ray microtomography. This article presents the details of destructive processes in the early stages of the deformation of reinforced polymers under uniaxial stretching, investigated at the micro level. Individual structural elements of the composite–filaments, parallel fiber bundles, the nonuniformity of the polymer binder distribution, and continuity defects—were observed under an external load. We have considered the influence of the material architecture and technological defects on fracture evolution in cross-ply and quasi-isotropic fiber-reinforced plastics. The results indicate the sequence of irreversible structural changes before the destruction of the material.

## 1. Introduction

Fiber-reinforced plastics are fundamental for modern technologies in aviation and aerospace engineering and the automobile industry. They are widely employed due to their notable properties, specifically their low weight and favorable mechanical data, which originate from the CFRP material architecture [1,2,3]. Carbon-fiber-reinforced polymers consist of fibers of 4–8 µm diameter that are assembled into threads and prepreg plies of 70–200 µm thickness [4]. The regular arrangement of fibers impregnated with an epoxy binder forms plies stacked into CFRP composites of diverse types depending on their orientation—unidirectional, cross-ply, or quasi-isotropic. There are high levels of elasticity and strength anisotropy in carbon plies. Experiments show that the elastic modulus along the fibers is appreciably higher than across them, and the polymer matrix possesses substantially lower strength compared with the fibers [4,5]. The properties of plies, their mutual orientation in stacks, and the parameters of interlayer bonding describe the mechanical properties of CFRP materials as a whole. Modern technologies in the hands of engineers enable the creation of high-strength materials with specified properties and anisotropy. Current problems involve minimizing technological defects in the material, controlling the quality of adhesive contacts between structural components in the composite, and studying the relations between structural features and mechanical properties. These problems can be solved with the help of high-resolution nondestructive imaging techniques developed for the detection and identification of damage mechanisms in material under a load.

The assessment and monitoring of bulk microstructure evolution in fiber-reinforced composites under external mechanical or thermo-mechanical loading is a topical problem in reinforced polymer science, especially for studying the processes of the irreversible deformation and fracture of reinforced polymers. These processes in such materials cannot be reduced to pure fragile or plastic behavior. As the load increases, the intricate material architecture causes a series of complicated multistage fracture processes. The destruction starts from microscopic defects such as cracks in the polymer matrix and detachments at fiber–polymer borders. Under higher loads, the number of defects increases and their size expands, while growth stops at the layer interfaces and initiates the delamination of the plies. Finally, because of the fractures, the fibers break and the sample loses its integrity when the applied load P achieves its critical value P_max_. This final stage is observed in the shortest interval of the loading range, 5–10% of P_max_. So, the early and intermediate stages of deformation are of special interest from the viewpoint of the lifetime of the material.

Nondestructive visualization methods are capable of monitoring the real-time evolution of structural changes under a load. Usually, optical microscopy, atomic-force microscopy (AFM), and scanning electron microscopy (SEM) techniques produce images of a rather smooth sample surface and require thin slices that need special preparation, leading to microstructural damage. To study processes of structural changes, destructive techniques require a large number of serial measurements of similar samples. Existing techniques for the nondestructive testing (NDT) of composite materials [6,7,8,9] are dedicated to industrial applications with low spatial resolution. Currently, the high spatial resolution needed for studying the microscopic structure of reinforced composites is provided only by techniques such as impulse acoustic microscopy [10,11,12,13] and X-ray computer microtomography (micro-CT) [14,15,16,17].

Micro-CT studies of CFRP material can be performed with X-ray tubes (in cone-beam geometry) or synchrotron radiation. Micro-CT allows the production of multiscale 3D images with a high spatial resolution, down to submicron values, which is necessary for monitoring microstructural changes in the early stages of deformation. Micro-CT layouts based on both types of X-ray sources provide similar spatial resolutions, but the use of the synchrotron source enables the application of novel coherent techniques, such as phase-contrast imaging. In addition, laboratory X-ray microtomography requires much longer sessions of CT scanning.

Thus, in 2005, a laboratory micro-CT system was used for the 3D visualization of small voids and cracks induced by an external tensile load in glass and carbon CFRP laminates [18]. Synchrotron radiation X-ray tomography (SR micro-CT) was successfully applied for the investigation of the behavior of fiber fractures inside unidirectional quartz glass composites [19]. It should be noted that micro-CT has proven to be an effective tool for this kind of investigation, and a significant number of papers have been published in the last decade [20,21,22,23,24,25,26,27,28,29,30,31,32].

Micro-CT experiments are usually carried out with small (~mm) samples of CFRP laminate materials containing 4–7 plies. Breaking a thin sample apart after its loading into narrow matchstick samples [20] is one way to obtain a small volume of the material. Another way is the fabrication of a notched thin sample by mounting it in a special stress testing mini-machine fixed for in situ measurements on the micro-CT stage. Notched samples were used in most experiments [21,22,23,24,25,26,27,28,29,30,31]. For example, the occurrence of longitudinal and transverse cracks in 0° and 90° plies of loaded cross-ply CFRP samples was studied using the micro-CT technique in [19,21]. Experimental results allow one to describe the micromechanical mechanisms of crack nucleation in CFRPs and to develop mathematical models of this process [23]. A complex experimental and theoretical approach allowed for studying fiber rupture at the final stage of CFRP fracture [24,25,26] as well as estimating the influence of voids inside the composite bulk on cracks and fiber break formation [27]. In [28,29], X-ray micro-CT was applied to reveal microstructural disorders induced by fatigue testing. In [30], SR micro-CT helped to establish the main idea of the failure distribution caused by impact damage tolerance, interlaminar fracture toughness, and fiber fracture and tensile strength.

Recent results of SR micro-CT in studies on woven CFRP materials were compiled by A. Rashidi et al. [31]. The investigation of CFRP laminates with nanoscale reinforcement in polymer binders and damage progression therein under mechanical loading are presented in [32]. The assessment of the packing accuracy, including the fiber’s waviness [33] and misalignment, using micro-CT can be found in [34]. Experiments allow the validation of mathematical models used in polymer science for describing fracture processes in reinforced composites and the assessment of their lifetime [22,25,35,36].

This paper aims to describe the dynamics of structural changes in cross-ply CFRP laminate under a tensile load using high-resolution SR micro-CT and to reveal the main mechanisms of composite destruction, which is of primary interest in the estimation of the material’s lifetime, by taking into account the effect of the initial structural defects.

## 2. Materials and Methods

**Figure 1:**
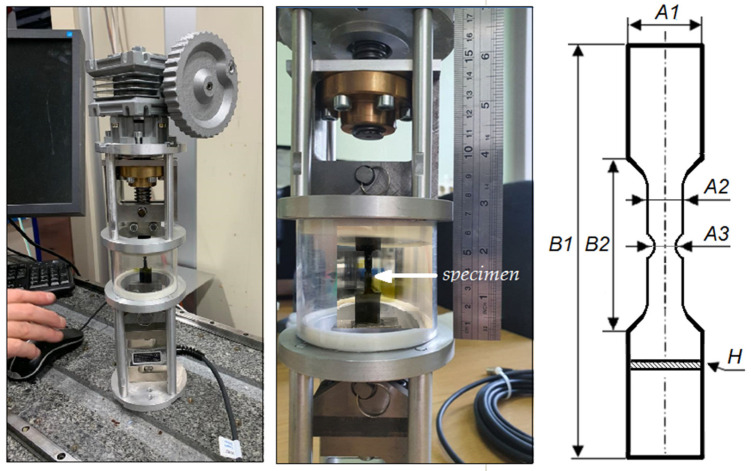
Photo of the tensile machine for in situ X-ray tomography study of samples under load (**left**). The setup and drawing of the CFRP sample (**right**).

The CFRP samples under investigation were made from carbon fiber/epoxy prepreg ACM 1208–C200UD (Aksa A38–3K/ACM 1208) manufactured by UMATEX, Moscow, Russia. The typical autoclave curing conditions for porosity-free laminates were a full vacuum of 1 bar and an autoclave pressure of 7 bar. All samples were selected from the same panel. The prepreg ply thickness was 0.2 ± 0.01 mm, and the samples were made of 4 plies with the cross-ply orientation of fibers [0/90/90/0]. The nominal thickness (H) of samples was 0.82 ± 0.01 mm. They were cut using a diamond cutting tool in the shape presented in Figure 1 and Table 1.

The experimental setup for the CFRP sample tensile test was a system of clamps with a vertical arrangement of samples. One of them was fixed on a linear mobility unit to apply a force to the sample. The other clamp was attached to a tensometric sensor to measure the force. The overall dimensions of the device were 10 cm × 40 cm, and the weight was 5 kg. The device included an electronic unit and software based on the ZETLAB product for the monitoring and registration of the loading curve. The miniature vertical tensile machine was designed to produce a tension force up to 3500 N. The strain of the sample under tension was measured via a direct contact method using an optoelectronic sensor LIR-DA7 with a resolution of 0.5 microns.

Synchrotron radiation micro-CT (SR micro-CT) is known to serve as an accurate nondestructive tool for the 3D investigation of CFRP materials. In this study, SR micro-CT experiments were carried out with the SYRMEP beamline (Elettra Synchrotron, Trieste, Italy) [37] using a pink X-ray beam with a mean energy of 23.6 keV and exposure time of 100 ms. The sample–detector distance was set at 165 mm. Images were obtained with an sCMOS camera (Hamamatsu C11440–22C–Flash 4.0 v.2, Hamamatsu Photonics K.K., Hamamatsu, Japan) with an effective pixel size of 2 µm. The CT scans were obtained with 3600 projections covering a total angle range of 360° in double-extended FOV mode. After properly stitching twin sinograms, the reconstruction procedure was performed. Data preprocessing and tomographic reconstruction were performed using the SYRMEP Tomo Project ver.1.6.3 (open source, https://github.com/ElettraSciComp/STP–GUI) [38] through the Filtered Back Projection algorithm, with the X-ray projection images preprocessed to eliminate or mitigate artifacts from experimental conditions and computational reconstruction. Ring artifacts were removed by an improved frequency-filtering method.

## 3. Results

Preliminary testing of the loaded CFRP samples was carried out for the evaluation of the threshold load corresponding to the loss of integrity. The value of the threshold load was measured to be 1135 ± 50 MPa. At a loading of 1035 ± 50 MPa, there was audible crepitation, probably accompanying fiber breaks. Since the purpose of this work was to observe the destruction processes in the composite at the early and intermediate stages of deformation, loading was restricted to 865 MPa (~75% of the threshold value) in all micro-CT stage studies.

**Figure 2:**
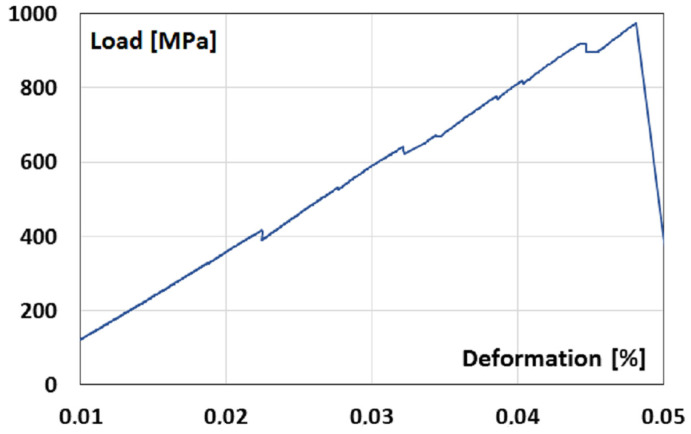
The load curve for the four-layer sample with the fiber orientation [0/90/90/0]. The fibers of the outer layers are oriented along a direction of external loading.

CFRP samples were stretched in steps from 38 to 865 MPa with a step of 190 MPa. The load curve in Figure 2 shows the process of brittle fracture with clear signs of load redistribution (notches on the curve). The average Young’s modulus was measured to be E = P/ε = 18.5 GPa in the range from 14.78 to 20.25 GPa.

A 3D study of the internal structure of the sample was performed in situ after the application of each step of the mechanical load for the localization and identification of irreversible deformations in the early stages. In this case, all cracks remain open enough for reliable detection in X-ray images. The first SR micro-CT measurement was carried out at 38 MPa when the initial preload provided both the minimal tension of the sample and the appropriate stability of the entire mechanical system with a low impact of errors and backlashes of sample fixation. A CT scan was performed by turning the whole stretching machine around its vertical axis, which provided data for the 3D visualization of a volume equal to 3 × 3 × 4 mm^3^. The visualization results are presented in next section.

### Microstructural Investigation

**Figure 3:**
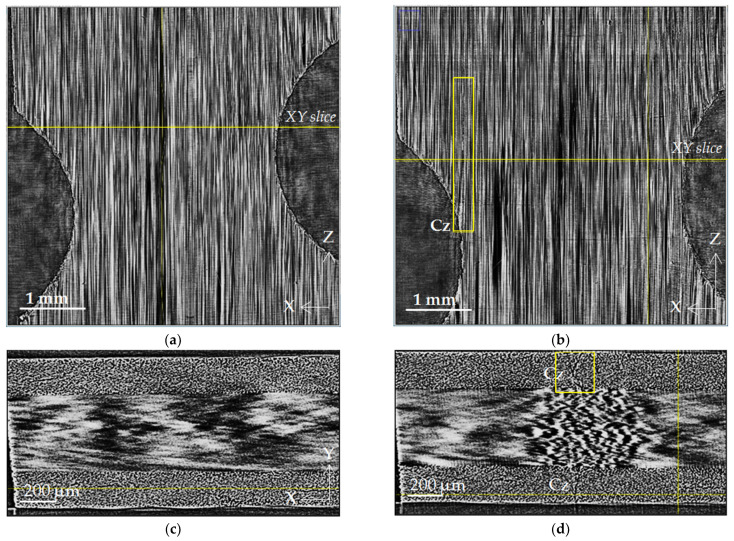
Microstructure of cross-ply CFR plastic in the [0°] layer: (**a**) initial state, 38 MPa; (**b**) loaded state, 865 MPa; (**c**,**d**) XY slices of the initial and loaded states, located as indicated in Figure 3a,b. Cz designates cracks spreading along the Z-axis, and K marks the kinking of the fiber package. The tension load was applied along the Z-axis.

**Figure 4:**
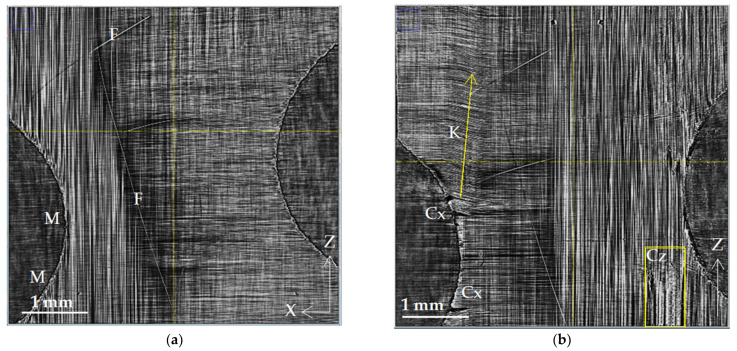
Microstructure of cross-ply CFR plastic at the interface of adjacent [0/90] layers: (**a**) initial state, 38 MPa; (**b**) loaded state, 865 MPa. The designations are M—mechanical damage; F—individual filament; Cx—crack spreading along the X-axis; Cz—crack along the Z-axis; and K—kinking of the fiber package.

**Figure 5:**
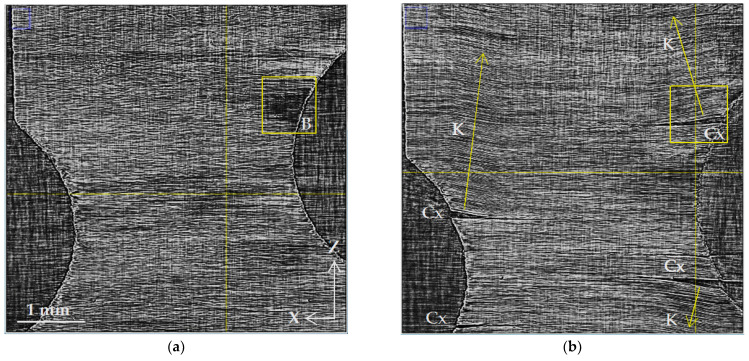
Microstructure of cross-ply CFR plastic at the interface of adjacent [90/90] layers: (**a**) initial state, 38 MPa; (**b**) loaded state, 865 MPa. B—binder pocket; Cx—crack spreading along the X-axis; K—kinking of fiber package.

**Figure 6:**
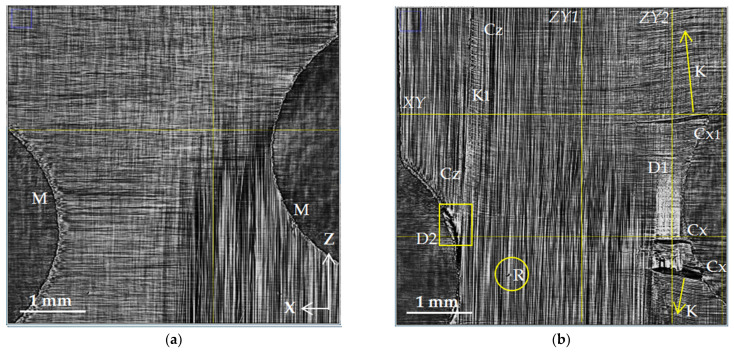
Microstructure of cross-ply CFR plastic at the interface of adjacent [90/0] layers: (**a**) initial state, 38 MPa; (**b**) loaded state, 865 MPa; M—mechanical damage after cutting; Cx—transverse crack spreading along the X-axis; K, K_1_—kinking of fibers; D—interlayer delamination; R—fiber rupture; XY, YZ1, and YZ2—positions of the orthogonal X-ray sections presented in Figure 8 and Figure 9.

**Figure 7:**
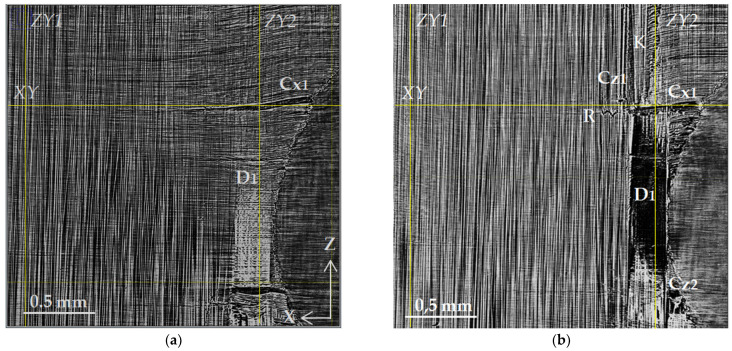
Magnified images of the damage zone D1: (**a**) layer presented in Figure 6b and (**b**) layer 25 µm deeper. D1 is interlayer delamination; Cx1—transverse crack spreading along the X-axis (90° fibers); Cz1 and Cz2—vertical cracks along the Z-axis (0° fibers); K—kinking of fibers; R—fiber rupture; XY—position of XY plane; ZY1 and ZY2—positions of YZ planes presented in Figure 8 and Figure 9.

The dynamics of microstructural changes inside the volume of CFRP samples were studied by gradually increasing the load from 38 to 865 MPa. Figure 3, Figure 4, Figure 5, Figure 6 and Figure 7 show the reconstructed SR micro-CT slices in the XZ plane obtained for two load states: (a) the initial microstructure at 38 MPa and (b) the deformed microstructure at 865 MPa. Axis Z corresponds to the direction of the applied load along the sample length, and axes X and Y are directed along the sample width and thickness, respectively (see Figure 1). All images demonstrate the microstructure resulting from the unidirectional stacking of carbon fibers inside the layers (Figure 3), while it becomes bidirectional at the interfaces of mutually perpendicular layers (Figure 4, Figure 5 and Figure 6).

Against the background of the regular arrangement of the fibers, the X-ray tomographic image of the top layer (Figure 3b) reveals subtle longitudinal cracks (Cz) directed along the Z-axis of the external load. Such microstructural damage Cz is even clearer in the XY slices (Figure 3d).

Figure 4a,b show the structure of the composite at the interface between the outer layers with the [0°] fiber orientation and the inner reinforcing layers with the [90°] orientation. In addition to the regular arrangement of fibers, the X-ray images show technological defects (F) in the form of separate stray fibers (fiber bundles). It is also necessary to note local microscopic damage (M) along the edge of the notch, which arose as a result of machine cutting during edge production. Figure 4b shows the evolution of these microscopic defects in the form of multiple Cx cracks propagating along the fibers and across them. In addition to cracks inside the [90°] layers oriented along the fibers (Cx), the images show extensively damaged areas (K) with the deformation of the parallel arrangement of the fibers. These areas are limited by the sharp bending of the fibers. The arrangement of fibers inside them turns out to be parallel to the contour of the crack. The deviation of the direction of the fibers from the original is determined by the geometry of the crack and its position relative to the sample fixation clamps, the magnitude, and the vector of the applied force.

Figure 5 shows the structure at the interface between the inner layers of [90°] fibers packed perpendicular to the external load. Despite strong artifacts resulting from X-ray coherent scattering under the grazing irradiation of the fibers in these layers, the SR micro-CT image enables us to recognize the principal deformation and defects of the microstructure. The evolution of multiple Cx cracks formed by tensile forces can be observed clearly at the interface between the layers. The location of the cracks mostly coincides with visible damage at the previous layer boundary (Figure 4b). The observed cracks appear to propagate throughout the entire depth of the second layer with openings at both boundaries of this inner layer. In the image of the initial structure (Figure 5a), there is a small dark zone measuring 0.5 × 0.5 mm^2^ (B) that corresponds to a local decrease in X-ray absorption and, consequently, to an excess of binder material of about 10 µm thickness near this layer boundary. When stretched, new damages appear in this zone (Figure 5b) in addition to Cx cracks, and the images show fractures and other local deformation areas of fiber stacking (K).

Figure 6 presents the formation of delamination at the interface with different fiber orientations (boundaries of the third [90°] and fourth [0°] layers). Cracks and delamination arise near the edges of the notch as a result of microdamage in the initial microstructure after machine cutting (M, cutting microdefects). The right part of Figure 6b shows that many visible transverse cracks (Cx) in the 90° layer transform into an extended interlayer delamination (D1). On the left side, in the 0° layer, several longitudinal cracks (Cz) are visible, which also lead to the growth of a small delamination (D2) at the layer boundary. The formation of transverse cracks (Cx) is accompanied by the curving or kinking of the fibers. The resulting deformation of the fibers is marked by K in the image. The formation of a longitudinal crack (Cz) also distorts fiber packing in 90° layers (K1). Since the load is significant, the defects also include fiber breaks (R).

The structure of the damaged zone D1 can be examined in more detail using high-resolution images (Figure 7). Figure 7a presents an enlarged view of zone D1 taken from Figure 6b. Figure 7b shows the microstructure in the tomographic slice 24 µm below. The upper edge of the delamination is clearly visible in Figure 7a as a white area due to the increased average density of fibers. The analysis of neighboring slices suggests the presence of a cavity. In Figure 7b, the presence of this discontinuity is shown as a dark area. The markers point out the transverse crack Cx1 along the axis in the 90° layer and vertical cracks Cz1 and Cz2 along the Z-axis in the 0° layer. There is also the bending of fibers (K) along the longitudinal crack Cz1. In the area of the intersection of two cracks, Cx1 and Cz1, along the delamination boundary D1, fiber rupture (R) occurred in the 0° layer. The representation of the fracture mechanism in this area can be clarified by orthogonal views in the XY and YZ planes, with the positions of these planes (XY, ZY1, and ZY2) shown in Figure 7.

**Figure 8:**
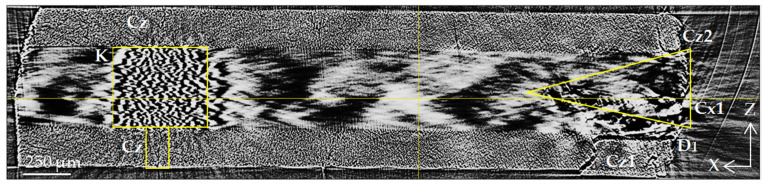
Transverse XY slice of the loaded CFRP sample (slice position is marked in Figure 6 and Figure 7). D1 is interlayer delamination; Cx—transverse crack spreading along the X-axis (90° fibers); Cz—vertical cracks along the Z-axis (0° fibers); K—kinking of fibers.

Figure 8 shows an XY plane slice with four layers of fiber orientations [0/90/90/0] visible. One can see a strong delamination D1 at the interface of layers 3 and 4 on the right side of the image. Near the delamination, there is a wide Cz crack running along the fibers across the 0° layer. There is also a similar Cz crack in the upper 0° layer. Extensive cracking Cx1 occurs in the 90° layers and expands to a considerable depth. As was mentioned above, nonlinear X-ray scattering complicates the interpretation of the SR micro-CT results for 90° layers; nevertheless, some important conclusions can still be drawn. On the left edge of the image, longitudinal cracks (Cz) in 0° layers are visible, which is accompanied by the deformation of the regular packing of fibers in 90° layers, and small ripples (K) are observed.

**Figure 9:**
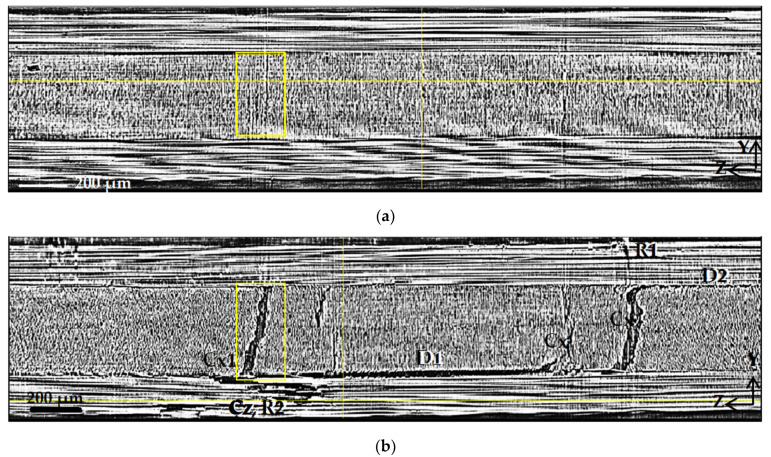
Transverse ZY1 slices (**a**) and ZY2 (**b**) marked in Figure 6 and Figure 7. D1 and D2 indicate delamination at interfaces 3–4 and 1–2, respectively; Cx—transverse cracks spreading along the X-axis in 90° plies, Cx1—crack indicated in Figure 6 and Figure 7; Cz—vertical cracks along the Z-axis in 0° plies; R1 and R2—rupture of fibers.

Planes ZY1 and ZY2 marked by lines in Figure 6 are presented in Figure 9.

Since the main damage is concentrated closer to the edge of the sample (see Figure 6, Figure 7 and Figure 8), the longitudinal ZY1 slice, positioned at a significant distance of 1.75 mm from the edge of the sample neck, demonstrates numerous thin longitudinal cracks (Cx) in the 90° layers. One of the cracks boxed in Figure 9a has been identified as the beginning of the large transverse crack Cx1 described in Figure 6, Figure 7 and Figure 8. The analysis of the full set of SR micro-CT experimental data indicates the deep penetration of transverse cracks in the 90° layers of the sample. In particular, the Cx1 crack spreads for over 1.75 mm from the edge surface to the depth of the sample.

Figure 9b shows the ZY2 section near the right edge of the sample. Transverse cracks in 90° layers are visible as large damages, with the opening width being in the range from 50 to 100 µm. One can see that at a certain size and width of the opening, intralayer Cx cracks spread across the boundaries of the layers and induce extensive delamination (D1 and D2) at these boundaries. In addition, longitudinal Cz cracks in 0° layers grow and contribute to the development of delamination. This process is also accompanied by local fiber breaks, as can be seen in zones R1 and R2.

## 4. Discussion

The process of the destruction of CFRP material is divided into several stages. The initial and intermediate stages are associated with the occurrence of damage in the binder material and at the interface between the binder and the reinforcing elements [1,39]. The isolated breaks of individual fibers are also visible at such moderate loads; massive fiber rupture takes place in the final stage of loading. A significant part of experimental research in the field of SR micro-CT is devoted to the observation of fiber breaks in the final stage of composite destruction [21,22,23,24,26]. In contrast to these works, the experimental results obtained in the current investigation are mainly focused on the study of processes in the volume of a polymer binder under the action of a tensile load. The results of a structural comparison before and after loading show the occurrence of a significant number of cracks: transverse Cx in 90° layers and longitudinal Cz (splits) in 0° layers. The beginning of fracture processes is usually associated with the formation of transverse cracks in layers orthogonal to the direction of the load [1,36]. The occurrence of longitudinal cracks along the fibers has also been observed and described earlier in microtomographic experiments [19,20,21,22,23,24]. Both longitudinal and transverse cracks occur more often on the lateral edges of the coupon, and in the developed state, they have maximum disclosure near the edges. This phenomenon can be caused both by the initial defects that occur when cutting samples and by the stress concentration near the edges [40]. Cracks spread deep into the volume of the corresponding layers. With a sufficient load, their access to the interface between the layers provides critical shear stress at the boundary and causes the occurrence of interlayer delamination. We observed such delamination (D) in our experiments on a loaded sample in the form of extended regions in XZ sections (Figure 6 and Figure 7) and in the form of dark bands along interlayer boundaries in XY and YZ sections (Figure 8 and Figure 9).

One of the natural results of tensile loading is the rupture of fibers. This is also observed in our X-ray images—elements designated by the letter R (Figure 7). With the moderate value of the applied load, only individual fiber breaks are visible in most images. However, in the YZ cross-section (Figure 9b) of the lower 0° layer, an extensive longitudinal crack (Cz) is observed, including the rupture of a significant part of the fibers passing through region R2.

This part of the defective microstructure representation corresponds to the generally accepted scheme of the irreversible deformation of CFRP materials. Due to micro-CT investigations [19,20,21,22,23,24,32], the formation of longitudinal cracks along fibers in layers with a 0° orientation and their contribution to the process of composite destruction were revealed. The role of this type of damage has been confirmed by the results of ultrasound studies [5].

A new type of microstructural violation has been revealed in our experiments. The formation of extensive areas of fiber contortion in regions adjacent to the developed longitudinal and transverse cracks has been observed. The buckling and kinking of fiber bundles are unusual for tensile testing; they are considered classical violations under compression [16,41]. Nevertheless, the tomographic images demonstrate the occurrence of regions with the distorted fiber package. The layers with a 90° orientation are the most vulnerable in terms of elasticity since the load falls on the matrix. Transverse cracks begin to form from the edges of the sample and grow deep into the material along the fibers. The crack opening angle depends on the magnitude of the applied load and varies from the maximum value at the sample edge to zero at the crack tip, where the integrity of the matrix is still preserved. Under a tensile load, the crack edges move farther and farther apart. Geometrically, the fiber package follows the crack outlines. Reinforcing fibers curve and give extensive areas (strips K) of contorted parallel fiber packing above and below the crack. Still, the value of the bending deformation of the fibers is insufficient for their destruction [42]. In the studied cross-ply samples, the opening and growth of cracks in 90° layers contribute to an uneven redistribution of tension and fiber rupture in the adjacent 0° layers. An area of contorted parallel packing is observed in Figure 6b and Figure 7b above the crack Cx1, repeating the outline of the upper edge of the crack. The curvature of fibers spreads up, forming a strip (K) of the distorted structure. The same track is visible in Figure 6b below the crack Cx.

Another type of distorted structure occurs near longitudinal cracks resulting from the splitting of fibers in layers with zero orientation. In Figure 6b, near the longitudinal crack (Cz) in the 0° layer, a narrow vertical track K1 of inclined fibers in the adjacent 90° layer is visible. These deformations (fiber kinking) are caused, apparently, by the effect of transverse compressive stresses σ_x_ arising in the 0° layer under the action of the tensile load σ_z_.

The presence of distorted fiber packing in 90° layers is also displayed in XY tomograms. The large-scale distribution of light and dark spots conveys the wavy packing of parallel fibers in the inner 90° layers in Figure 3d and Figure 8. However, the representation of the distorted fiber package is displayed in XY slices as areas with small-scale ripples (Figure 3d and Figure 8).

Distorted fiber packing causes a complex stress distribution in the zone of fiber contortion, especially near the interply interface. In Figure 7b, we can see the fiber rupture zone R in the 0° layer that is tight against fiber buckling in the adjacent layer with a 90° orientation. This fiber rupture zone, as well as the kink zone K1 in Figure 6b, demonstrates the importance of taking into account the forces caused by fiber distortion under external loads. The revealed features of the processes of the irreversible deformation and destruction of carbon fiber plastics—the formation of longitudinal and transverse cracks with finite opening and the occurrence of fiber buckling and kinking zones—suggest the need to refine the micromechanical models that are currently used for the mathematical modeling of loading processes and evaluate the resources of CFRP composite materials and structures [43].

The given X-ray images, primarily XZ slices, can serve as a basis for characterizing the uniformity of the distribution of reinforcing fibers, the degree of their parallelism, the identification of disordered fibers and their bundles, and so on. Heterogeneity in the distribution of stacked fibers in the 0° layer (Figure 3a), a dark area with an increased binder content (Figure 5a), and several separate undirected fibers (Figure 4) are structural imperfections that characterize the technology used for sample preparation.

## 5. Conclusions

The SR micro-CT technique allowed us to present a detailed description of the early and intermediate stages of the fractural process for a cross-ply [0/90] s composite to provide data for the validation and improvement of micromechanical models, which are currently used to evaluate composite materials and structure resources.

The results of a comparison of the microstructures before and after loading show longitudinal splits (Cz) in 0° layers and transverse cracks (Cx) in 90° layers spreading from the edges towards the center of the sample. As the load increases, the cracks reach the layer interfaces and trigger interlayer delamination growth. The rupture of fibers is also observed in our X-ray images.

The curvature and kinking of the fibers are new types of microstructural changes that have been found in our experiments. The source of kinking is expanded cracks, both longitudinal and transverse. These deformations appear to be induced by the transverse compressive stress σ_x_ arising in the 0° layer under the tensile load σ_z_.

## Figures and Tables

**Table 1:** Dimensions of the CFRP specimens in the mechanical tests. Accuracy is ± 0.01 mm.

A1	A2	A3	B1	B2	H
15.00	5.00	3.18	100.00	20.00	0.82

## Data Availability

Not applicable.
